# Characterization and phylogenetic analysis of the complete chloroplast genome of *Ophiorrhiza pumila* (Rubiaceae)

**DOI:** 10.1080/23802359.2021.1925985

**Published:** 2021-06-14

**Authors:** Qikai Huang, Guoyin Kai

**Affiliations:** College of pharmacy, Laboratory of Medicinal Plant Biotechnology, Zhejiang Chinese Medical University, Hangzhou, PR China

**Keywords:** *Ophiorrhiza pumila*, chloroplast genome, phylogenetic analysis

## Abstract

*Ophiorrhiza pumila* (Rubiaceae) is an herbaceous plant that grows streamside in forest gullies or wetlands in the shade. Complete chloroplast genome of *O. pumila* was obtained and analyzed its phylogeny relationship within Rubiaceae plants. The results showed that the genome had a typical quadripartite structure of 154,385 bp, and contained a total of 112 unique genes, including 79 protein-coding genes, 29 tRNA genes, and 4 rRNA genes. Phylogenetic analysis suggested that *O. pumila* is sister to a highly supported clade composed of 10 species including *Morinda officinalis*, *Gynochthodes cochinchinensis*, *Saprosma merrillii*, *Hedyotis ovata*, *Foonchewia guangdongensis*, *Dunnia sinensis*, *Paederia scandens*, *Leptodermis scabrida*, *Rubia cordifolia*, and *Galium mollugo*. The complete chloroplast genome provides valuable information for the phylogenetic analysis of *O. pumila.*

The genus *Ophiorrhiza* (Rubiaceae) widely distributed worldwide, consists of 321 species, 5 varieties, and 1 subspecies (Taher et al. 2020). Most of them are perennial herbs, approximately varying from 10 cm to 1 m in height. Some species such as *Ophiorrhiza pumila,* can accumulate camptothecin (CPT) in all tissues, which is used as a resource for anticancer medicine (Lee et al. 2020). *O. pumila* is a model plant used to study biosynthesis and regulation of monoterpene indole alkaloid, and is a sustainable source of CPT (Hao et al. 2021). It is distributed in southern Japan, southern China, northern Vietnam, and the Philippines, and naturally grows in forest gullies streamside and in wetlands in the shade (Liu et al. 2020). Due to morphological similarities among *Ophiorrhiza*, whole plastomes used for phylogenetic analysis and identification become more significant. Chloroplast, a common organelle related to photosynthesis in plant cells, has been reported to be associated with the synthesis of vitamins, pigments, fatty acids, and amino acids through various biochemical pathways. Chloroplast genome is conserved throughout higher plants at the structural and genic level, and some genes such as *matk*, *rbcl*, and *ndhF* were often used as DNA barcodes (Mehmood, Abdullah Shahzadi, et al. 2020; Mehmood, Abdullah Ubaid, Bao, et al. 2020; Mehmood, Abdullah Ubaid, Shahzadi, et al. 2020). In this study, the complete chloroplast (cp) genome of *O. pumila* (GenBank accession number: MW528277) was sequenced and reported.

Fresh young sample of *O. pumila* was obtained from aseptic seedlings cultivated in a plant growth chamber at Zhejiang Chinese Medical University, Hangzhou, Zhejiang, China (30°4′59″N, 119°53′31″E). Total genomic DNA was extracted with a modified cetyltrimethyl ammonium bromide (CTAB) method (Doyle and Doyle 1986). The voucher specimens (No. DX-3_180794) of *O. pumila* were preserved in the Laboratory of Medicinal Plant Biotechnology. The extracted DNA was sheared into 300–400 bp fragments with a Covaris M220 (Covaris, Woburn, MA), and a shotgun library was built following the procedure of NEB Next^®^ UltraTM DNA Library Prep Kit for Illumina (NEB) (US). The library was paired-end sequenced on the Illumina HiSeq 4000 platform. With the cp genome of *Dunnia sinensis* (GenBank accession MN883829) as a reference sequence, *O. pumila* chloroplast genome reads from the Illumina sequencing data adopting the BLAST method were selected. The reads were assembled using SOPAdenovo version 2 (Shenzhen, China) with k-mer = 39 and default parameters, and scaffolds were obtained (Luo et al. 2012). Then the scaffolds were used as seed sequences to finish the cp genome sequence by NOVOPlasty (Dierckxsens et al. 2017) with the following settings: k-mer = 37, type = mito, insert size = 350, and other parameters as default. New complete circular chloroplast genomes of *O. pumila* were yielded with a quadripartite structure sequence 154,385 bp in length. Similar to other angiosperms (Gao et al. 2018), the new genome consisted of a pair of inverted repeats (IRs), a large single copy (LSC), and small single copy (SSC) regions of 26,067, 84,101, and 18,150 bp in length, respectively. The GC content of the *O. pumila* cp genome was 37.7%, and the corresponding values in the LSC, SSC, and IR regions were 35.6%, 31.7%, and 43.2%, respectively. Gene annotation of the *O. pumila* cp genome was performed using the web application GeSeq with the *D. sinensis* cp genome as a reference sequence (https://chlorobox.mpimp-golm.mpg.de/geseq.html) and then manually edited by Geneious version 10.3 (Auckland, New Zealand) (Kearse et al. 2012). The cp genome contained 133 genes, including 88 protein-coding genes, 37 tRNA genes, and 8 rRNA genes. Of these, 112 were unique, and nine protein-coding genes, eight tRNA genes, and four rRNA genes were duplicated in IR regions. Among them, 12 unique genes contained one intron, three unique genes (*rps12*, *ycf3*, and *clpP*) contained two introns, and the rest are intronless. IRscope (Amiryousefi et al. 2018) was used to visualize the structure of the IR/SC borders, the *rpl22* gene spanned the LSC/IRb region with 366 bp in LSC region and 102 bp in the IRa region. The *ycf1* gene spanned the SSC/IRa region with 4468 bp in the SSC region and 1136 bp in the IRa region. The *ycfl* gene in the IRb/SSC junction was an incomplete duplication of the normal functional copy of *ycfl* in the IRa/SSC junction, which is a phenomenon that is often found in choroplasts (Liu et al. 2018).

The phylogenetic tree (Figure 1) of 22 species of Rubiaceae and one Valerianaceae species was constructed using Mega-X version 10.0.5 software (US) (Kumar et al. 2018) with a gamma distributed (G) model and 1000 bootstrap replicates. All the 23 complete cp genomes were aligned by MAFFT software (Katoh and Standley 2013). According to the results, *O. pumila* was close to a clade consisting of 10 species (*Morinda officinalis*, *Gynochthodes cochinchinensis*, *Saprosma merrillii*, *Hedyotis ovata*, *Foonchewia guangdongensis*, *Dunnia sinensis*, *Paederia scandens*, *Leptodermis scabrida*, *Rubia cordifolia*, and *Galium mollugo*). The cp genome provides valuable information for the phylogenetic analysis of *O. pumila.*

**Figure 1.**
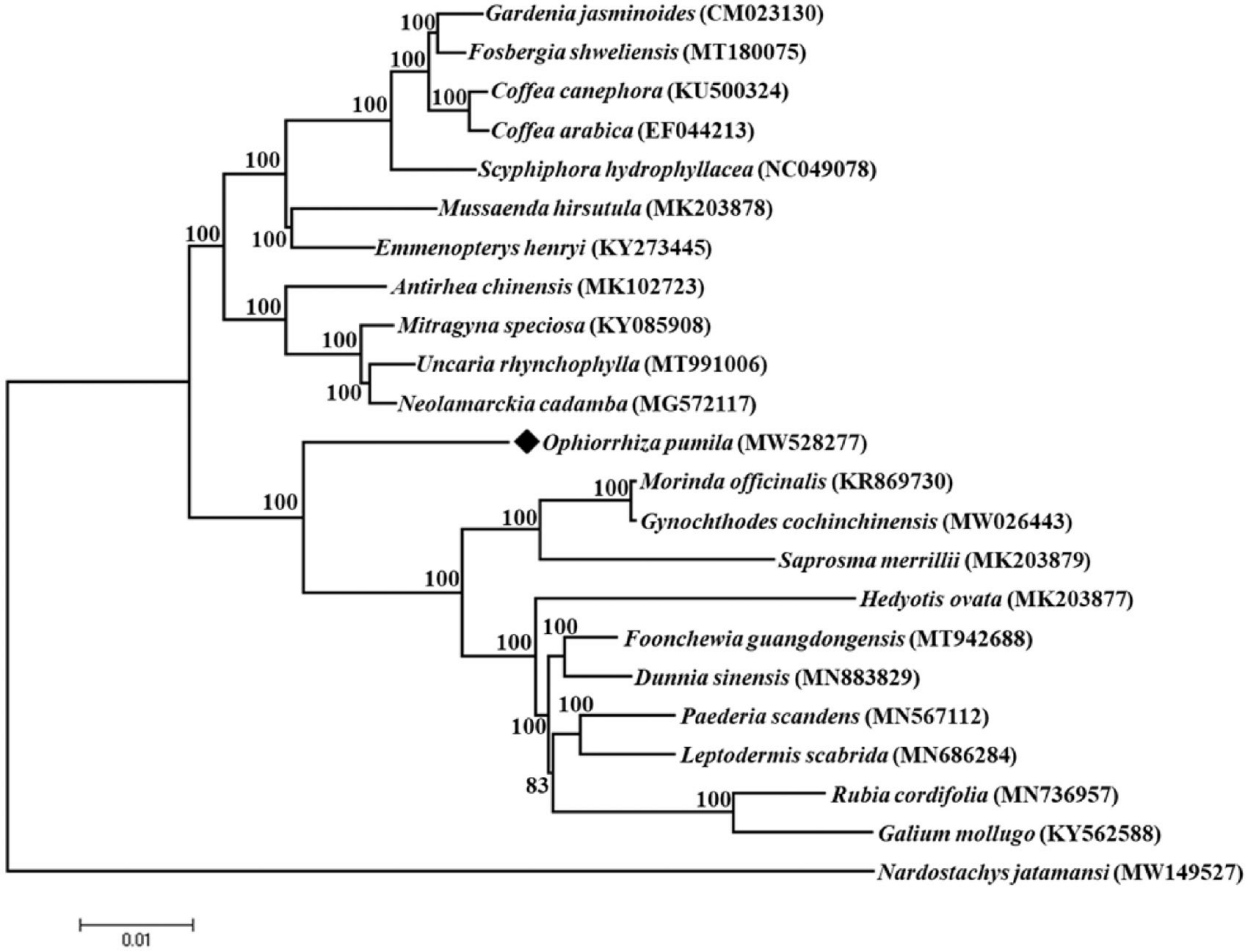
The maximum likelihood (ML) phylogenetic tree of 23 Rubiales species chloroplast genomes.

## Data Availability

The data that support the findings of this study are openly available under accession number MW528277 in GenBank of National Center for Biotechnology Information at https://www.ncbi.nlm.nih.gov.
